# Functional network centrality indicates interactions between APOE4 and age across the clinical spectrum of AD

**DOI:** 10.1016/j.nicl.2024.103635

**Published:** 2024-06-24

**Authors:** Aïda B. Fall, Maria Giulia Preti, Mohamed Eshmawey, Sonja M. Kagerer, Dimitri Van De Ville, Paul G. Unschuld, for the Alzheimer’s Disease Neuroimaging Initiative

**Affiliations:** a Faculty of Medicine, University of Geneva (UNIGE), Geneva, Switzerland; b Geriatric Psychiatry Service, University Hospitals of Geneva (HUG), Thônex, Switzerland; c CIBM Center for Biomedical Imaging, Switzerland; d Neuro-X Institute, École Polytechnique Fédérale de Lausanne, Geneva, Switzerland; e Institute for Regenerative Medicine (IREM), University of Zurich, Zurich, Switzerland; f Psychogeriatric Medicine, Psychiatric University Hospital Zurich (PUK), Zurich, Switzerland

**Keywords:** Alzheimer’s disease, Apolipoprotein E4, Brain age, Functional connectivity, Eigenvector centrality, Genetics

## Abstract

Advanced age is the most important risk factor for Alzheimer’s disease (AD), and carrier-status of the Apolipoprotein E4 (APOE4) allele is the strongest known genetic risk factor. Many studies have consistently shown a link between APOE4 and synaptic dysfunction, possibly reflecting pathologically accelerated biological aging in persons at risk for AD.

To test the hypothesis that distinct functional connectivity patterns characterize APOE4 carriers across the clinical spectrum of AD, we investigated 128 resting state functional Magnetic Resonance Imaging (fMRI) datasets from the Alzheimer’s Disease Neuroimaging Initiative database (ADNI), representing all disease stages from cognitive normal to clinical dementia. Brain region centralities within functional networks, computed as eigenvector centrality, were tested for multivariate associations with chronological age, APOE4 carrier status and clinical stage (as well as their interactions) by partial least square analysis (PLSC).

By PLSC analysis two distinct brain activity patterns could be identified, which reflected interactive effects of age, APOE4 and clinical disease stage. A first component including sensorimotor regions and parietal regions correlated with age and AD clinical stage (p < 0.001). A second component focused on medial-frontal regions and was specifically related to the interaction between age and APOE4 (p = 0.032).

Our findings are consistent with earlier reports on altered network connectivity in APOE4 carriers. Results of our study highlight promise of graph-theory based network centrality to identify brain connectivity linked to genetic risk, clinical stage and age. Our data suggest the existence of brain network activity patterns that characterize APOE4 carriers across clinical stages of AD.

## Highlights

• Distinct FC patterns characterize APOE4 carriers across clinical stages of AD.
• Graph theory-based network centrality identifies brain connectivity linked to APOE4.
• Network centrality can be used as biomarker for rTMS and tACS treatment responses.

## 1 Introduction

Sporadic Alzheimer’s disease (AD) is the most common cause for dementia at old age (Scheltens et al., 2021). AD includes a continuum of clinical disease stages, with multiple clinical phenotypes converging to a final common endpoint of severe cognitive disorder (Aisen et al., 2017, Devi and Scheltens, 2018). A plethora of studies have documented both brain structural and functional changes to be associated with aging (Kolb et al., 2003), allowing for a biological diagnosis of AD based on biomarkers linked to pathogenesis (Knopman et al., 2018, Jack et al., 2016). Synaptic dysfunction plays a central role in progression of AD, and consistent patterns of network dysfunction have been reported for all disease stages (Franzmeier et al., 2024, Wang et al., 2024, Buckner et al., 2009). In the presence of AD, pathological brain changes are characterized by the accumulation of neurotoxic waste products of neural activation; i.e., the proteins Amyloid-beta and tau (Craig-Schapiro et al., 2009). This occurs with a gradual deterioration of cognitive skills, often accompanied by emotional disorder, eventually leading to dementia. An altered organization of brain functional networks is a very early finding in AD, typically preceding structural loss of brain tissue (Joo et al., 2015).

Functional magnetic resonance imaging (fMRI) has proven to be a valid tool for assessing the disruption of functional brain networks in a context of AD related neuropathology (Dennis and Thompson, 2014, Zhang et al., 2022, Quevenco et al., 2017, Quevenco et al., 2019, Kagerer et al., 2020). This technique measures blood-oxygen-level dependent (BOLD) signals that indirectly reflect in vivo neuronal activity (Ogawa S et al., 1990). A synchronous fluctuation of BOLD signals in different brain areas, generally computed by Pearson’s correlation, is commonly referred to as functional connectivity (Friston et al., 1993). The computation of FC in resting-state acquisitions allowed to highlight the so-called resting-state networks, including the default mode network (DMN), which represents a signature of the resting-state condition, in opposition to task-positive networks. Alterations of the DMN have been proven both in healthy aging (Wen et al., 2020) and in different stages of AD (Greicius et al., 2004). Different methods have been developed to characterize functional connectivity and the properties of the obtained brain networks, for example by means of graph-based metrics, one of them is the eigenvector centrality, that reveals how central a region is within a network (Lohmann et al., 2010). These metrics have previously proven useful in unveiling alterations of functional connectivity in presence of subjective cognitive decline, mild cognitive impairment and AD (Binnewijzend et al., 2014, Sanz-Arigita et al., 2010, Qiu et al., 2016, Dai et al., 2019, Deng et al., 2021).

The presence of Apolipoprotein E4 (APOE4) allele, in contrast to the other two polymorphic alleles E2 and E3, has been shown to accelerate aging effects at the brain level (Deary et al., 2002) and increase the risk of developing AD (Wadhwani et al., 2019, James Bryan and Bennett, 2019, Serrano-Pozo et al., 2021). Moreover, the E4 isoform of the ApoE protein exhibits diminished efficiency in clearing Aβ in comparison to E2 and E3 variants, leading to accelerated aggregation of plaques (Wink et al., 2018, Wink et al., 2018, Yu et al., 2014, Risacher et al., 2015). Researchers reported functional brain alterations in young, healthy carriers of the APOE4 allele, implying that these variant influences neuronal activity before disease manifestation (Filippini et al., 2009, Su et al., 2017, Zheng et al., 2018). In older E4 carriers, decreased activity was observed in the frontal and temporal lobes, the cerebellum, and subcortical brain regions (Filippini et al., 2011, Ma et al., 2017, Sheline et al., 2010, Machulda et al., 2011, Cai et al., 2017, Luo et al., 2017). Despite this, there remains limited understanding of the distinct impacts of the APOE genotype on brain aging. This line of work represents a critical domain for enhancing our comprehension of Alzheimer's disease pathology and its detection.

A series of studies have focused on the progression of clinical symptoms and cognitive decline in MCI and AD, suggesting a spectrum with shared neuropathology characterized by impaired resting-state brain network connectivity, with accelerated progression in APOE4 carriers (Sperling et al., 2010, Kanai et al., 1999, Wang et al., 2017, Karcher et al., 2020, Sanabria-Diaz et al., 2021, Sanabria-Diaz et al., 2021). The aim of our study was to use graph- theory based network centrality analysis to identify APOE4 associated brain activity patterns across the clinical spectrum of AD, as represented by different disease stages.

## 2 Methods

### 2.1 Participants

All our data came from the Alzheimer's Disease Neuroimaging Initiative (ADNI) database (adni.loni.usc.edu). For our study, we included 128 subjects, 76 non-symptomatic (39 Cognitive Normal (CN), 37 Subjective Memory Complaint (SMC)) and 52 symptomatic individuals (39 Mild Cognitive Impairment (MCI), 13 AD) (demographics reported in Table 1). We computed non-parametric testing to evaluate potential differences between groups regarding age, MMSE and education. The Kruskal-Wallis test results indicated no significant differences across these variables (p > 0.05). Additionally, there were also no significant differences in functional connectivity in females and males subjects, in both groups (APOE4 vs APOE4 non-carriers, p > 0.05). Inclusion criteria were availability of fMRI and T1 weighted images with acquisitions made with the same acquisition protocol on the same scanner as well as availability of clinical assessment, demographic data and APOE genotyping. Within our sample, 54 individuals are APOE4 carriers (genotypes E4/E4, E4/E3 and E4/E2) and 74 individuals are APOE4 non-carriers (genotypes E3/E3 and E3/E2). The genotype was analyzed from DNA samples of each participant with an APOE genotyping kit. Demographic information of our sample can be found in the results section (Table 1). The study was approved by the Institutional Review Boards of all the participating institutions of ADNI, and informed written consent was obtained from all participants.

**Table 1:** Demographics of our study population.

Demographics					
	APOE4 carriers		APOE4 non-carriers		All Subjects
	Symptomatic N = 27	Non-Symptomatic N = 27	Symptomatic N = 25	Non-Symptomatic N = 49	N = 128
Age	Mean = 72.28 std = 7.76	Mean = 71.69 std = 7.46	Mean = 74.26 std = 7.4	Mean = 73.29 std = 7.5	Mean = 73.42 std = 7.59
Sex	F/M = 13/14	F/M = 17/10	F/M = 13/12	F/M = 28/21	F/M = 71/57
MMSE	Mean = 27.85 std = 2.72	Mean = 27.84 std = 2.77	Mean = 28.26 std = 2.48	Mean = 28.28 std = 2.6	Mean = 28.09 std = 2.58
Education	Mean = 15.16 std = 2.3	Mean = 16.86 std = 2.25	Mean = 16.03 std = 2.64	Mean = 16.7 std = 2.16	Mean = 16.3 std = 2.39

N: number of subjects; std: standard deviation; F: Female; M: Male; MMSE: Mini-Mental State Examination; There were no significant differences between APOE4 vs non-APOE4 carriers in any of these demographic variables.

### 2.2 Clinical assessments

All clinical phenotyping, including diagnosis, was performed as reported in the ADNI protocol (https://adni.loni.usc.edu/wp-content/themes/freshnews-dev-v2/documents/clinical/ADNI3_Protocol.pdf). The cognitive controls were individuals without significant impairment in cognitive functions or activities of daily living and without any signs of depression. SMC subjects reported problems with memory without objective cognitive impairments and without any signs of depression. MCI individuals presented clear evidence of cognitive impairment, small or no functional impairment in daily living activities and with cognitive concern reported by the patient and/or family. Finally, AD individuals were established as MCI but with impairment in daily life activities.

The exclusion criteria were any significant neurologic disease, such as Parkinson’s disease, multi-infarct dementia, Huntington’s disease, brain tumor, epileptic seizure disorder, normal pressure hydrocephalus, progressive supranuclear palsy, subdural hematoma, multiple sclerosis, or history of significant head trauma followed by persistent neurologic deficits or known structural brain abnormalities.

### 2.3 Data acquisition

All study participants received neuroimaging on 3 Tesla MRI instruments, using standardized sequences according to the ADNI MR neuroimaging protocol (https://adni.loni.usc.edu/methods/documents/mri-protocols/). The resting-state fMRI images were acquired using an echo-planar imaging (EPI) sequence (repetition time (TR) = 3000 ms, echo time (TE) = 30 ms, flip angle = 90°, number of slices = 197, voxel size = 3.4 mm × 3.4 mm × 3.4 mm, slice thickness = 3.4 mm, and voxel matrix = 448 × 448, scan duration = 10 min). The anatomical images were acquired with an MPRAGE sequence (repetition time (TR) = 2300 ms, echo time (TE) = 3 ms, flip angle = 9°, voxel size = 1 mm × 1 mm × 1 mm, slice thickness = 1 mm).

### 2.4 Imaging preprocessing

Both anatomical and functional images were pre-processed with a standardized in-house-developed preprocessing pipeline (Richiardi et al., 2012) implemented in MATLAB (MATLAB 2021a version 9.10; MathWorks Inc., Natick, MA, USA) and using functions from SPM8 and SPM12 (https://www.fil.io-n.ucl.ac.uk/spm/). Briefly, this included volume realignment and spatial smoothing (FWHM = 5 mm), as well as nuisance signal removal (six motion parameters, linear and quadratic trends, average white matter, and cerebrospinal fluid signals).

After registration of anatomical images to the functional individual space, functional brain images were parcellated using an atlas containing 379 brain regions, including 360 cortical (Glasser et al., 2016) and 19 subcortical ones (Fischl et al., 2002). Individual gray matter (GM) maps were extracted for each subject, and intersected with the registered atlas parcellation, to extract regional BOLD time series, computed by averaging the signal over all GM voxels in each atlas region. These were band-pass filtered with a cut-off of 0.01–0.15 Hz to focus on typical resting-state fluctuations.

### 2.5 Functional connectivity computation

To compute FC for each subject, we calculated pairwise Pearson’s correlations between pre-processed regional time series, yielding a 379 × 379 symmetric matrix per individual.

We then computed eigenvector centrality, a graph metric that quantifies the connectivity of brain regions within a network. This metric weighs each brain region based on its centrality in the overall network (Lohmann et al., 2010). It therefore assigns higher centrality values to regions that are connected to other regions that are themselves highly connected (hubs). This metric allows to identify key brain regions that serve as major hubs in the network and therefore play crucial roles in brain function. It is computed as the first eigenvector of the FC matrix, which corresponds to the largest eigenvalue. It therefore represents a lower rank (rank-1) approximation of the FC. As a result, we obtained 379 variables, each representing the eigenvector centrality of a distinct brain region, for each subject. These variables were then used for further statistical analysis.

### 2.6 Statistical analysis

Statistical analysis was performed in MATLAB (2021a version 9.10) by using in-house code and functions from the Statistics and Machine Learning toolbox. We investigated the relationship between eigenvector centrality, age and the presence of APOE4 with Partial Least Square Correlation (PLSC) analysis (Krishnan et al., 2011), specifically designed to explore multivariate relationships between a brain and a behavioral set of variables. In our case, the brain data matrix × included regional eigenvector centrality values (379 values) for each subject, and the behavioral matrix Y included the following six variables: (1) age of each subject; (2) presence of APOE4 (0/1); (3) presence of objective cognitive symptoms, coded as 1 for symptomatic (AD and MCI); and 0 for non-symptomatic individuals (controls and individuals with subjective memory complaints); (4) interaction between age and the presence of APOE4; (5) interaction between age and cognitive symptoms; (6) interaction between presence of APOE4 and cognitive symptoms. The three behavioral variables (1–3) were z-scored across the population (Krishnan et al., 2011), while interactions (4–6) were computed as a multiplication between z-scored variables. By singular value decomposition (SVD) of the covariance matrix between X and Y, PLSC extrapolates sets of variables (left and right singular vectors of the SVD) that maximize the correlation between brain and behavior; i.e. the brain saliences V and behavioral saliences U. As in the original PLCS formulation (Krishnan et al., 2011), SVD components are tested for significance by permutation testing on singular values (1000 permutations). Brain and behavioral saliences include patterns of either brain regions, or behavioral variables that maximize covariance between the brain and behavioral dataset, expressed by singular values (Krishnan et al., 2011). Stable elements in brain and behavioral saliences are assessed via bootstrapping (1000 samples), performed to compute standard errors for these metrics. The corresponding bootstrap ratios were thresholded at an absolute value greater than 2, which corresponds to a 95 % confidence interval and implies a stable contribution of the variables to the correlation (Baracchini et al., 2023). Brain and behavioral scores, also referred to as PLS latent variables, are then computed as linear combinations of the original data weighted by PLS saliences; that is, Lx = XV and Ly = YU, respectively. These scores allow to visualize how subjects are projected in the PLS space optimized for brain-behavior covariance.

In addition, an *aging score* Ly^age^ = Y^age^ * U^age^ is introduced here, with Y^age^ and U^age^ defined as subsets of the original Y and U matrices containing only the age-related behavioral variables and saliences (that is, age, interaction age-E4 and interaction age-symptoms). This can be seen therefore as a behavioral score specific to age-related variables; i.e., a linear combination of age, APOE4-age interaction and symptoms-age interaction weighted by their behavioral saliences. This score, computed for each subject, allows us to evaluate how each individual is affected by age effects on brain connectivity, and if this contribution is different with respect to the presence of APOE4 or cognitive symptoms.

## 3 Results

### 3.1 Age and AD symptoms correlate with decreased centrality in somatomotor regions

**Fig. 1:**
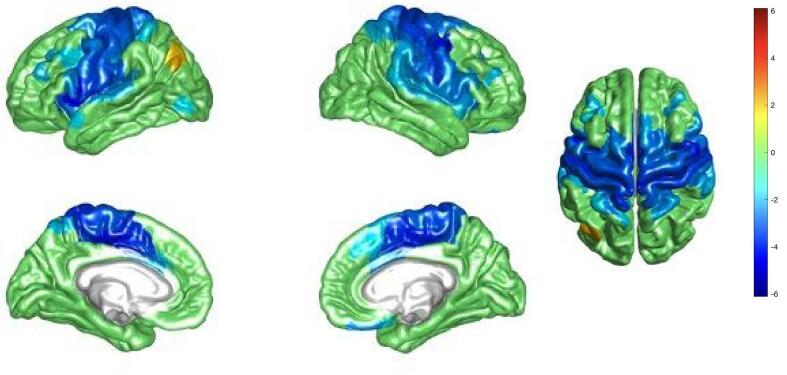
Brain plot representing the first PLS brain salience pattern of correlation driven by age, the presence of APOE4 and cognitive symptoms. We can observe a decreased centrality in the somatomotor and ventral attention networks (blue) and an increased centrality in the left inferior parietal area (orange) correlated with age, the presence of APOE4 and cognitive symptoms. The values are bootstrap ratios thresholded at the absolute value of 2, corresponding to a 95% confidence interval. (For interpretation of the references to colour in this figure legend, the reader is referred to the web version of this article.)

**Fig. 2:**
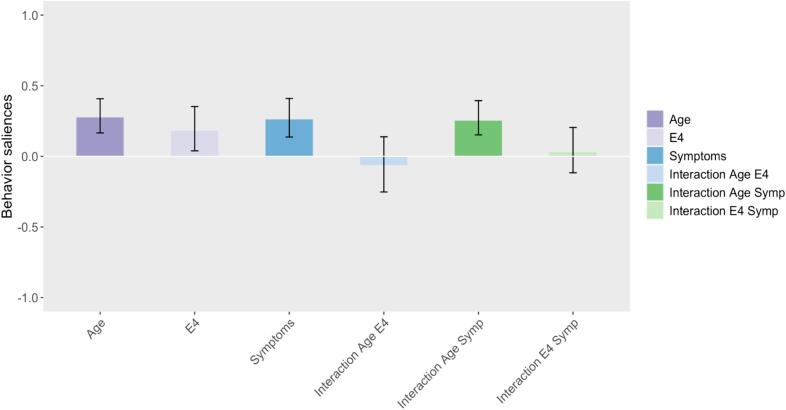
Bar plot representing the behavioral saliences of the first PLS component, indicating significant contribution of age, the presence of APOE4 and cognitive symptoms, as well as the interaction between age and symptoms, to the brain-behavior correlation. The bars correspond to the confidence interval 95%.

PLSC analysis yielded two significant latent components. By looking at pairs of brain and behavioral saliences of each component (for example shown for the first component in Fig. 1, Fig. 2, respectively), we can highlight which brain regions and behavioral variables contribute the most to the multivariate correlation. The brain-behavior scores trajectories can be visualized in Supplementary Fig. 7 for the first component and in Supplementary Fig. 8 for the second component. When interpreting behavioral saliences, the ones whose confidence intervals do not cross the zero are retained to significantly contribute to the multivariate correlation (Krishnan et al., 2011).

The first component (p-val < 0.001) highlights a significant relationship between decreased centrality in dorsal sensorimotor and ventral attention networks (Fig. 1, dark blue areas), and the three behavioral variables (age, the presence of APOE4 and cognitive symptoms), as well as the interaction between age and symptoms (Fig. 2). Indeed, age and symptoms appear to dominate this component, with stronger influence on the correlation pattern (higher behavioral saliences for the behavioral variables (1), (3) and (5)). This observation is further supported by Supplementary Fig. 7, where it is apparent that individuals exhibiting cognitive symptoms predominantly contribute to the observed brain pattern, as shown by their scores diverging drastically from zero compared to other subjects. We also found opposite correlation behavior in a small region within the left inferior parietal region (orange), where increased centrality is linked with the same behavioral variables (age, the presence of APOE4, cognitive symptoms and the interaction between age and symptoms).

### 3.2 Age with APOE4 affects brain centrality in a specific orbitofrontal-parietal pattern independently of the presence of cognitive symptoms

**Fig. 3:**
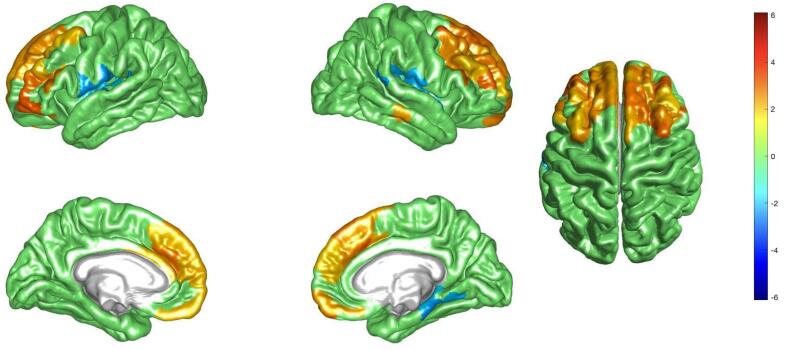
Brain plot representing the second PLS brain salience pattern of correlation driven by APOE4 and its interaction with age. We can observe an increased centrality in the frontal lobes (light orange) and a decreased centrality in the left posterior opercular as well as the right insular and frontal opercular (light blue) that is correlated with age and the presence of APOE4. The values are z-scored and thresholded at a bootstrap ratio of 2. (For interpretation of the references to colour in this figure legend, the reader is referred to the web version of this article.)

**Fig. 4:**
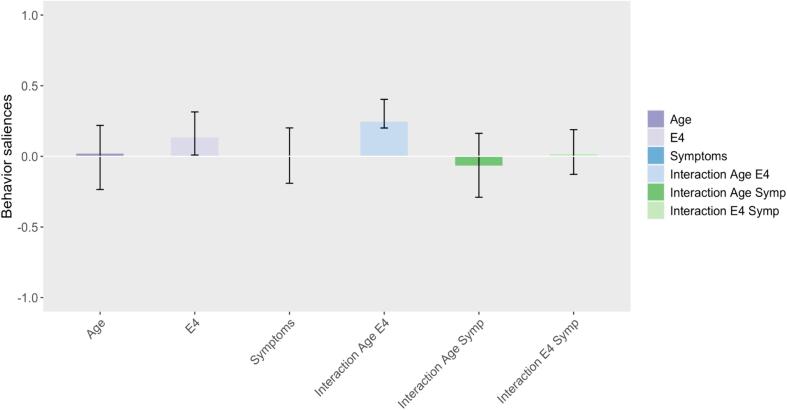
Bar plot representing the PLS behavioral salience, capturing multivariate correlation between APOE4, age-APOE4 interaction and the eigenvector centrality in the brain salience pattern shown in Fig. 3. The bars correspond to the confidence interval 95 %.

The second PLS component (p-val = 0.032) appears particularly interesting for us, as it highlights, in a totally data-driven way, a distinct correlation pattern, specific to only age and APOE4, and not to the presence of cognitive symptoms. These highlighted brain regions belonging to default mode and frontoparietal networks. In particular, increased centrality in extended regions of the medial frontal area (Fig. 3, light orange) and decreased centrality in a small focused areas in the left posterior opercular as well as the right insular and frontal opercular (light blue), is associated with the interaction between age and the presence of the APOE4, independently of the cognitive symptoms. We can see in fact from Fig. 4 that only the age-APOE4 interaction (behavioral variable (4)), and in a minor proportion APOE4 (behavioral variable (2)), contribute to the significant brain-behavior correlation. This observation is further illustrated in Supplementary Fig. 8 where the brain-behavior correlation appears more prominently driven by APOE4 carriers.

### 3.3 Age effects on the brain are different in presence of APOE4 or cognitive symptoms

**Fig. 5:**
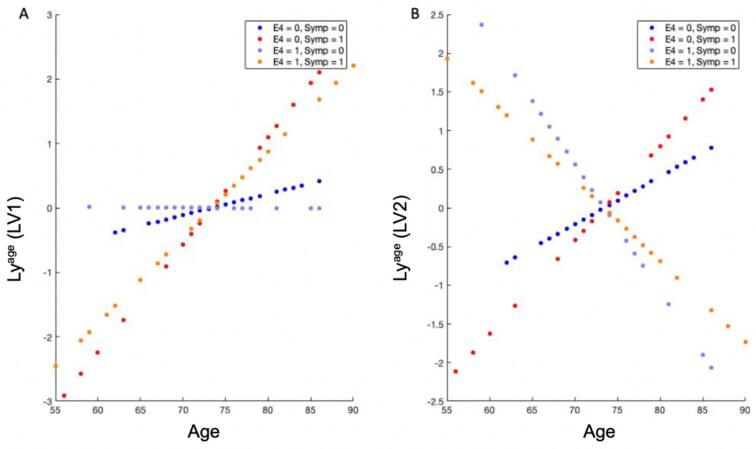
Aging score for the first PLS component projected on chronological age and colored by APOE4 carrier status and cognitive symptoms index. We observed an effect of both the presence of cognitive symptoms and APOE4, more pronounced for the presence of cognitive symptoms. *B)* Aging score for the second PLS component projected on chronological age and colored by APOE4 carrier status and by cognitive symptoms index. We observed an effect of both the presence of cognitive symptoms and APOE4, but much more pronounced for the presence of APOE4, in fact there is a clear opposition between having or not the genetic risk factor for Alzheimer’s disease.

To better interpret each subject’s contribution in the brain-age correlation patterns and how it differs in the presence of APOE4 and cognitive symptoms, we plotted the computed aging score projected on the chronological age of subjects (Figs. 5A and B for the two PLS components, respectively). For both components, subjects group in linear patterns with distinct slopes depending on the presence of symptoms and/or APOE4 genetic risk factor. In other words, subjects are differently affected in terms of brain age effects, depending on if they are APOE4 carriers or not, and if they present AD-related cognitive symptoms or not. However, for the first component (Fig. 5A), the difference in the distribution is more pronounced between presence or absence of cognitive symptoms, confirming the fact that age effects on somatomotor and ventral attention circuits (highlighted by the first brain salience) are mainly driven by the presence of cognitive symptoms.

For the second component (Fig. 5B), instead, the major effect is given by the presence or absence of APOE4, as shown by a clear opposition between subjects with or without the genetic risk factor for Alzheimer’s disease. This further confirms that age-related effects on centralities in frontal regions (as depicted by the second brain salience pattern in Fig. 3) are specific to APOE4 carriers.

## 4 Discussion

The presence of FC alterations in aging is largely explored in literature with a plethora of different methods (Cassady et al., 2019, Dennis and Thompson, 2014, Vij et al., 2018, Wen et al., 2020). In this study, we used graph theory to characterize FC in AD, as reported earlier (Yu et al., 2021, Brier et al., 2014, Sanz-Arigita et al., 2010). Our approach included a graph-based metric called eigenvector centrality (Lohmann et al., 2010, Wink et al., 2018, Wink et al., 2018). To our knowledge, our results are the first published evidence on the presence of functional connectivity (FC) patterns linked to the interaction between APOE4 and aging, which remains stable across the clinical spectrum of Alzheimer’s disease stages.

We found a first signature brain pattern associated with age, the presence of cognitive symptoms as well as the presence of APOE4, corroborating the evidence of neurodegenerative brain alterations. Brain regions involved showed decreased centrality as an effect attributable to age, cognitive symptoms and APOE4, and were part of both somatomotor and ventral attention networks, which is consistent with earlier fMRI data on aging and Alzheimer’s disease (Li et al., 2011). In particular, several studies have documented altered functional connectivity patterns within the somatomotor network in AD. Specifically, reduced centrality and disrupted connectivity have been observed, indicating compromised motor function and sensorimotor integration (Dennis and Thompson, 2014, Wang et al., 2015, Devanand et al., 2008, Verghese et al., 2007). These alterations may contribute to the motor symptoms commonly observed in AD patients such as slowed movement and decreased spontaneous activity (Cerejeira et al., 2012, Tosto et al., 2015). Furthermore, previous studies have shown increased tau and amyloid deposition associated with APOE4 status in brain regions within both the somatomotor and ventral attention networks (Drzezga et al., 2009, Ossenkoppele et al., 2016). Prior studies also indicate how attentional systems are affected (Li et al., 2011, Malhotra, 2019), besides the usual memory alteration manifested by AD individuals.

The aging score computed in our study was meant to better grasp the age contribution in the identified brain patterns and how this contribution differs with the presence of APOE4 or cognitive symptoms. We found that, for the first component, only the presence of cognitive symptoms seems to have an effect along with age.

In addition to this, we found a brain pattern indicating an opposition between frontal regions and insular areas to be *specifically* related to the effects of age in presence of APOE4, which was present in all clinical stages of AD. Presence of both activation and suppression of brain activity may reflect synaptic dysfunction and dysregulation associated with AD brain pathology, as suggested recently (Schreiner et al., 2024).

This observed brain pattern exhibits an increased centrality in frontal regions, alongside a decreased centrality in insular areas, associated with the interaction between age and the presence of APOE4. While previous studies have reported a reduced centrality in occipital regions among healthy APOE4 carriers (Wink et al., 2018), individuals with mild cognitive impairment (MCI) (Meinzer et al., 2012) and in AD populations (Binnewijzend et al., 2014, Sanz-Arigita et al., 2010, Rombouts et al., 2009), our findings diverge from this pattern. However, the increased centrality in the left anterior cingulate gyrus, correlated with the interaction between age and the presence of APOE4, aligns with prior research (Binnewijzend et al., 2014). Moreover, earlier research on structural connectivity has demonstrated increased local efficiency, a graph metric that reflects the efficiency of information flow among nearby brain regions, in the anterior cingulate gyrus of individuals with MCI and AD (Lin et al., 2019) as well as in subjective cognitive decline individuals (Xu et al., 2022). This underlines the novelty of our study, which employs eigenvector centrality as a sensitive tool to assess AD-related genetic risk, and specifically captures the interaction of this risk factor with aging. In fact, adopting a multivariate analysis framework allows us to consider all variables at the same time and successfully disentangle the effects of AD pathology and presence of APOE4. The distinction elucidates the specific influence of the APOE4 allele on brain connectivity patterns, irrespective of cognitive impairment severity. In essence, our findings delineate distinct brain patterns associated with APOE4 presence, regardless of cognitive symptomatology, thus offering valuable insights into the neurobiological mechanisms underlying AD-related genetic risk.

Increased BOLD signal observed in APOE4 carriers has been interpreted as a potential compensatory mechanism aimed at maintaining normal cognitive performance (Bondi et al., 2005, Dickerson et al., 2004, Han et al., 2007, Burggren and Brown, 2014). Conversely, reduced BOLD signal in E4 carriers has been linked to early pathological effects in individuals at genetic risk of developing AD (Borghesani et al., 2008, Adamson et al., 2011, Håglin et al., 2023). In light of these findings, we can postulate a similar scenario in our study, where the increased centrality associated with aging in APOE4 presence (red pattern in Fig. 3) may serve as a compensatory mechanism to preserve normal cognitive function despite presence of AD brain pathology, while decreased centrality in the right insular and frontal opercular brain regions could reflect local distress of affected neuronal populations. As such, the here characterized brain pattern might be used to develop response markers for disease modifying therapeutic intervention, with particular relevance for persons at increased risk for AD, as conferred by APOE4.

While repetitive transcranial magnetic stimulation (rTMS) and transcranial alternating current stimulation (tACS) represent promising approaches for patients with mild to moderate AD (Koch et al., 2022, Benussi et al., 2022), such novel therapeutic interventions might be targeted on network-dysregulation within regions of the pattern characterized in our present study.

Our sample includes three individuals with E4/E2 carrier-status. Excluding these subjects to have a homogeneous sample did not alter the current results. In a future study, which should include more subject for each genetic profile, ideally in a longitudinal setting, it would be interesting to see if specific brain connectivity modifications are present in individuals with APOE2, possibly protective for AD (Farrer et al., 1997), and investigate differences from APOE4 related brain patterns regarding implicated brain regions, but also for predictive, individual risk profiling.

## 5 Conclusion

Early detection and dynamic staging remain a crucial challenge in order to manage efficacious therapeutic intervention in AD. In this work, we explored the relationship between age on brain functional connectivity in AD and the potential mediation of this effect conferred by APOE4, as indicated by graph-theory based network centrality. We found a signature brain pattern associated with age and the presence of APOE4, corroborating evidence of synaptic dysfunction as a core neuropathological finding across Alzheimer’s disease stages. Future longitudinal studies are needed to validate our findings in a context of disease progression and possible use as response-markers in disease modulating therapeutic intervention.

## CRediT authorship contribution statement

**Aïda B. Fall:** Writing – original draft, Visualization, Methodology, Formal analysis, Data curation, Conceptualization. **Maria Giulia Preti:** Writing – review & editing, Supervision, Conceptualization. **Mohamed Eshmawey:** Writing – review & editing. **Sonja M. Kagerer:** Writing – review & editing. **Dimitri Van De Ville:** Supervision, Conceptualization, Writing – review & editing. **Paul G. Unschuld:** Writing – review & editing, Supervision, Conceptualization.

## Declaration of competing interest

The authors declare that they have no known competing financial interests or personal relationships that could have appeared to influence the work reported in this paper.

## Data Availability

The authors do not have permission to share data.

## References

[b0005] Adamson M.M., Hutchinson J.B., Shelton A.L., Wagner A.D., Taylor J.L. (2011). Reduced hippocampal activity during encoding in cognitively normal adults carrying the APOE ɛ4 allele. Neuropsychologia.

[b0010] Aisen P.S., Cummings J., Jack C.R., Morris J.C., Sperling R., Frölich L., Jones R.W., Dowsett S.A., Matthews B.R., Raskin J., Scheltens P., Dubois B. (2017). On the path to 2025: understanding the Alzheimer's disease continuum. Alzheimers Res. Ther..

[b0015] Baracchini G, Zhou Y, da Silva Castanheira J, Hansen JY, Rieck J, Turner GR, Grady CL, Misic B, Nomi J, Uddin LQ, Spreng RN. The biological role of local and global fMRI BOLD signal variability in human brain organization. bioRxiv [Preprint]. 2023 Oct 23:2023.10.22.563476. doi: 10.1101/2023.10.22.563476. PMID: 37961684; PMCID: PMC10634715. 10.1038/s41467-026-68700-0 PMC12960827 41618090

[b0020] Benussi A., Cantoni V., Grassi M., Brechet L., Michel C.M., Datta A., Thomas C., Gazzina S., Cotelli M.S., Bianchi M., Premi E., Gadola Y., Cotelli M., Pengo M., Perrone F., Scolaro M., Archetti S., Solje E., Padovani A., Pascual-Leone A., Borroni B. (2022). Increasing brain gamma activity improves episodic memory and restores cholinergic dysfunction in Alzheimer's disease. Ann. Neurol..

[b0025] Binnewijzend MA, Adriaanse SM, Van der Flier WM, Teunissen CE, de Munck JC, Stam CJ, Scheltens P, van Berckel BN, Barkhof F, Wink AM. Brain network alterations in Alzheimer's disease measured by eigenvector centrality in fMRI are related to cognition and CSF biomarkers. Hum Brain Mapp. 2014 May;35(5):2383-93. doi: 10.1002/hbm.22335. Epub 2013 Sep 3. PMID: 24039033; PMCID: PMC6869112. 10.1002/hbm.22335 PMC6869112 24039033

[b0030] Bondi M.W., Houston W.S., Eyler L.T., Brown G.G. (2005). fMRI evidence of compensatory mechanisms in older adults at genetic risk for Alzheimer disease. Neurology.

[b0035] Borghesani P.R., Johnson L.C., Shelton A.L., Peskind E.R., Aylward E.H., Schellenberg G.D., Cherrier M.M. (2008). Altered medial temporal lobe responses during visuospatial encoding in healthy APOE*4 carriers. Neurobiol. Aging.

[b0040] Brier MR, Thomas JB, Fagan AM, Hassenstab J, Holtzman DM, Benzinger TL, Morris JC, Ances BM. Functional connectivity and graph theory in preclinical Alzheimer's disease. Neurobiol Aging. 2014 Apr;35(4):757-68. doi: 10.1016/j.neurobiolaging.2013.10.081. Epub 2013 Oct 18. PMID: 24216223; PMCID: PMC3880636. 10.1016/j.neurobiolaging.2013.10.081 PMC3880636 24216223

[b0045] Buckner R.L., Sepulcre J., Talukdar T., Krienen F.M., Liu H., Hedden T., Andrews-Hanna J.R., Sperling R.A., Johnson K.A. (2009). Cortical hubs revealed by intrinsic functional connectivity: mapping, assessment of stability, and relation to Alzheimer's disease. J. Neurosci..

[b0050] Burggren A., Brown J. (2014). Imaging markers of structural and functional brain changes that precede cognitive symptoms in risk for Alzheimer's disease. Brain Imaging Behav..

[b0055] Cai S., Jiang Y., Wang Y., Wu X., Ren J., Lee M.S., Lee S., Huang L. (2017). Modulation on brain gray matter activity and white matter integrity by APOE ε4 risk gene in cognitively intact elderly: A multimodal neuroimaging study. Behav. Brain Res..

[b0060] Cassady K., Gagnon H., Lalwani P., Simmonite M., Foerster B., Park D., Peltier S.J., Petrou M., Taylor S.F., Weissman D.H., Seidler R.D., Polk T.A. (2019). Sensorimotor network segregation declines with age and is linked to GABA and to sensorimotor performance. Neuroimage.

[b0065] Cerejeira J., Lagarto L., Mukaetova-Ladinska E.B. (2012). Behavioral and psychological symptoms of dementia. Front. Neurol..

[b0070] Craig-Schapiro R, Fagan AM, Holtzman DM. Biomarkers of Alzheimer's disease. Neurobiol Dis. 2009 Aug;35(2):128-40. doi: 10.1016/j.nbd.2008.10.003. Epub 2008 Oct 28. PMID: 19010417; PMCID: PMC2747727. 10.1016/j.nbd.2008.10.003 PMC2747727 19010417

[b0075] Dai Z., Lin Q., Li T., Wang X., Yuan H., Yu X., He Y., Wang H. (2019). Disrupted structural and functional brain networks in Alzheimer's disease. Neurobiol. Aging.

[b0080] Deary I.J., Whiteman M.C., Pattie A., Starr J.M., Hayward C., Wright A.F., Carothers A., Whalley L.J. (2002). Cognitive change and the APOE epsilon 4 allele. Nature.

[b0085] Deng S., Sun L., Chen W., Liu X., Chen S. (2021). Effect of APOEε4 on functional brain network in patients with subjective cognitive decline: A resting state functional MRI Study. Int J Gen Med..

[b0090] Dennis E.L., Thompson P.M. (2014). Functional brain connectivity using fMRI in aging and Alzheimer's disease. Neuropsychol. Rev..

[b0095] Devanand DP, Liu X, Tabert MH, Pradhaban G, Cuasay K, Bell K, de Leon MJ, Doty RL, Stern Y, Pelton GH. Combining early markers strongly predicts conversion from mild cognitive impairment to Alzheimer's disease. Biol Psychiatry. 2008 Nov 15;64(10):871-9. doi: 10.1016/j.biopsych.2008.06.020. Epub 2008 Aug 23. PMID: 18723162; PMCID: PMC2613777. 10.1016/j.biopsych.2008.06.020 PMC2613777 18723162

[b0100] Devi G., Scheltens P. (2018). Heterogeneity of Alzheimer's disease: consequence for drug trials?. Alzheimers Res. Ther..

[b0105] Dickerson B.C., Salat D.H., Bates J.F., Atiya M., Killiany R.J., Greve D.N., Dale A.M., Stern C.E., Blacker D., Albert M.S., Sperling R.A. (2004). Medial temporal lobe function and structure in mild cognitive impairment. Ann. Neurol..

[b0110] Drzezga A., Grimmer T., Henriksen G., Mühlau M., Perneczky R., Miederer I., Praus C., Sorg C., Wohlschläger A., Riemenschneider M., Wester H.J., Foerstl H., Schwaiger M., Kurz A. (2009). Effect of APOE genotype on amyloid plaque load and gray matter volume in Alzheimer disease. Neurology.

[b0115] Farrer LA, Cupples LA, Haines JL, Hyman B, Kukull WA, Mayeux R, Myers RH, Pericak-Vance MA, Risch N, van Duijn CM. (1997) Effects of age, sex, and ethnicity on the association between apolipoprotein E genotype and Alzheimer disease. A meta-analysis. APOE and Alzheimer disease meta analysis consortium. JAMA. Oct 22-29;278(16):1349-56. PMID: 9343467. 9343467

[b0120] Filippini N., Ebmeier K.P., MacIntosh B.J., Trachtenberg A.J., Frisoni G.B., Wilcock G.K., Beckmann C.F., Smith S.M., Matthews P.M., Mackay C.E. (2011). Differential effects of the APOE genotype on brain function across the lifespan. Neuroimage.

[b0125] Filippini N, MacIntosh BJ, Hough MG, Goodwin GM, Frisoni GB, Smith SM, Matthews PM, Beckmann CF, Mackay CE. Distinct patterns of brain activity in young carriers of the APOE-epsilon4 allele. Proc Natl Acad Sci U S A. 2009 Apr 28;106(17):7209-14. doi: 10.1073/pnas.0811879106. Epub 2009 Apr 8. PMID: 19357304; PMCID: PMC2678478. 10.1073/pnas.0811879106 PMC2678478 19357304

[b0130] Fischl B., Salat D.H., Busa E., Albert M., Dieterich M., Haselgrove C., van der Kouwe A., Killiany R., Kennedy D., Klaveness S., Montillo A., Makris N., Rosen B., Dale A.M. (2002). (2002) Whole brain segmentation: automated labeling of neuroanatomical structures in the human brain. Neuron.

[b0135] Franzmeier N., Dehsarvi A., Steward A., Biel D., Dewenter A., Roemer S.N., Wagner F., Groß M., Brendel M., Moscoso A., Arunachalam P., Blennow K., Zetterberg H., Ewers M., Schöll M. (2024). Elevated CSF GAP-43 is associated with accelerated tau accumulation and spread in Alzheimer's disease. Nat. Commun..

[b0140] Friston K.J., Frith C.D., Liddle P.F., Frackowiak R.S. (1993). (1993) Functional connectivity: the principal-component analysis of large (PET) data sets. J. Cereb. Blood Flow Metab..

[b0145] Glasser MF, Coalson TS, Robinson EC, Hacker CD, Harwell J, Yacoub E, Ugurbil K, Andersson J, Beckmann CF, Jenkinson M, Smith SM, Van Essen DC. (2016) A multi-modal parcellation of human cerebral cortex. Nature. 2016 Aug 11;536(7615):171-178. doi: 10.1038/nature18933. Epub 2016 Jul 20. PMID: 27437579; PMCID: PMC4990127. 10.1038/nature18933 PMC4990127 27437579

[b0150] Greicius MD, Srivastava G, Reiss AL, Menon V. (2004) Default-mode network activity distinguishes Alzheimer's disease from healthy aging: evidence from functional MRI. Proc Natl Acad Sci U S A. 2004 Mar 30;101(13):4637-42. doi: 10.1073/pnas.0308627101. Epub 2004 Mar 15. PMID: 15070770; PMCID: PMC384799. 10.1073/pnas.0308627101 PMC384799 15070770

[b0155] Håglin S., Koch E., Schäfer Hackenhaar F., Nyberg L., Kauppi K. (2023). APOE ɛ4, but not polygenic Alzheimer's disease risk, is related to longitudinal decrease in hippocampal brain activity in non-demented individuals. Sci. Rep..

[b0160] Han SD, Houston WS, Jak AJ, Eyler LT, Nagel BJ, Fleisher AS, Brown GG, Corey-Bloom J, Salmon DP, Thal LJ, Bondi MW. Verbal paired-associate learning by APOE genotype in non-demented older adults: fMRI evidence of a right hemispheric compensatory response. Neurobiol Aging. 2007 Feb;28(2):238-47. doi: 10.1016/j.neurobiolaging.2005.12.013. Epub 2006 Jan 24. PMID: 16434125; PMCID: PMC1705815. 10.1016/j.neurobiolaging.2005.12.013 PMC1705815 16434125

[b0165] Jack CR Jr, Bennett DA, Blennow K, Carrillo MC, Feldman HH, Frisoni GB, Hampel H, Jagust WJ, Johnson KA, Knopman DS, Petersen RC, Scheltens P, Sperling RA, Dubois B. A/T/N: An unbiased descriptive classification scheme for Alzheimer disease biomarkers. Neurology. 2016 Aug 2;87(5):539-47. doi: 10.1212/WNL.0000000000002923. Epub 2016 Jul 1. PMID: 27371494; PMCID: PMC4970664. 10.1212/WNL.0000000000002923 PMC4970664 27371494

[b0170] James Bryan D., Bennett D.A. (2019). Causes and patterns of dementia: an update in the era of redefining Alzheimer's disease. Annu. Rev. Public Health.

[b0175] Joo SH, Lim HK, Lee CU. Three Large-Scale Functional Brain Networks from Resting-State Functional MRI in Subjects with Different Levels of Cognitive Impairment. Psychiatry Investig. 2016 Jan;13(1):1-7. doi: 10.4306/pi.2016.13.1.1. Epub 2015 Nov 20. PMID: 26766941; PMCID: PMC4701672. 10.4306/pi.2016.13.1.1 PMC4701672 26766941

[b0180] Kagerer S.M., van Bergen J.M.G., Li X., Quevenco F.C., Gietl A.F., Studer S., Treyer V., Meyer R., Kaufmann P.A., Nitsch R.M., van Zijl P.C.M., Hock C., Unschuld P.G. (2020). APOE4 moderates effects of cortical iron on synchronized default mode network activity in cognitively healthy old-aged adults. Alzheimers. Dement. (Amst)..

[b0185] Kanai M., Shizuka M., Urakami K., Matsubara E., Harigaya Y., Okamoto K., Shoji M. (1999). Apolipoprotein E4 accelerates dementia and increases cerebrospinal fluid tau levels in Alzheimer's disease. Neurosci. Lett..

[b0190] Karcher H., Savelieva M., Qi L., Hummel N., Caputo A., Risson V., Capkun G. (2020). Alzheimer's disease neuroimaging initiative. modelling decline in cognition to decline in function in Alzheimer's disease. Curr. Alzheimer Res..

[b0195] Knopman D.S., Haeberlein S.B., Carrillo M.C., Hendrix J.A., Kerchner G., Margolin R., Maruff P., Miller D.S., Tong G., Tome M.B., Murray M.E., Nelson P.T., Sano M., Mattsson N., Sultzer D.L., Montine T.J., Jack C.R., Kolb H., Petersen R.C., Vemuri P., Canniere M.Z., Schneider J.A., Resnick S.M., Romano G., van Harten A.C., Wolk D.A., Bain L.J., Siemers E. (2018). The National Institute on Aging and the Alzheimer's Association Research Framework for Alzheimer's disease: Perspectives from the Research Roundtable. Alzheimers Dement..

[b0200] Koch G., Casula E.P., Bonnì S., Borghi I., Assogna M., Minei M., Pellicciari M.C., Motta C., D'Acunto A., Porrazzini F., Maiella M., Ferrari C., Caltagirone C., Santarnecchi E., Bozzali M., Martorana A. (2022). (2022) Precuneus magnetic stimulation for Alzheimer's disease: a randomized, sham-controlled trial. Brain.

[b0205] Kolb B., Gibb R., Robinson T.E. (2003). “Brain plasticity and behavior” (PDF). Curr. Dir. Psychol. Sci..

[b0210] Krishnan A., Williams L.J., McIntosh A.R., Abdi H. (2011). Partial Least Squares (PLS) methods for neuroimaging: a tutorial and review. Neuroimage.

[b0215] Li C., Zheng J., Wang J., Gui L. (2011). (2011) Comparison between Alzheimer's disease and subcortical vascular dementia: attentional cortex study in functional magnetic resonance imaging. J. Int. Med. Res..

[b0220] Lin SY, Lin CP, Hsieh TJ, Lin CF, Chen SH, Chao YP, Chen YS, Hsu CC, Kuo LW. Multiparametric graph theoretical analysis reveals altered structural and functional network topology in Alzheimer's disease. Neuroimage Clin. 2019;22:101680. doi: 10.1016/j.nicl.2019.101680. Epub 2019 Jan 25. PMID: 30710870; PMCID: PMC6357901. 10.1016/j.nicl.2019.101680 PMC6357901 30710870

[b0225] Lohmann G., Margulies D.S., Horstmann A., Pleger B., Lepsien J., Goldhahn D., Schloegl H., Stumvoll M., Villringer A., Turner R. (2010). (2010) Eigenvector centrality mapping for analyzing connectivity patterns in fMRI data of the human brain. PLoS One.

[b0230] Luo X., Qiu T., Jia Y., Huang P., Xu X., Yu X., Shen Z., Jiaerken Y., Guan X., Zhou J., Zhang M. (2017). ADNI. Intrinsic functional connectivity alterations in cognitively intact elderly APOE ε4 carriers measured by eigenvector centrality mapping are related to cognition and CSF biomarkers: a preliminary study. Brain Imaging Behav..

[b0235] Ma C., Wang J., Zhang J., Chen K., Li X., Shu N., Chen Y., Liu Z., Zhang Z. (2017). Disrupted Brain Structural Connectivity: Pathological Interactions Between Genetic APOE ε4 Status and Developed MCI Condition. Mol. Neurobiol..

[b0240] Machulda MM, Jones DT, Vemuri P, McDade E, Avula R, Przybelski S, Boeve BF, Knopman DS, Petersen RC, Jack CR Jr. Effect of APOE ε4 status on intrinsic network connectivity in cognitively normal elderly subjects. Arch Neurol. 2011 Sep;68(9):1131-6. doi: 10.1001/archneurol.2011.108. Epub 2011 May 9. PMID: 21555604; PMCID: PMC3392960. 10.1001/archneurol.2011.108 PMC3392960 21555604

[b0245] Malhotra P.A. (2019). Impairments of attention in Alzheimer's disease. Curr. Opin. Psychol..

[b0250] Meinzer M., Antonenko D., Lindenberg R., Hetzer S., Ulm L., Avirame K., Flöel A. (2012). Electrical brain stimulation improves cogni- tive performance by modulating functional connectivity and task- specific activation. J. Neurosci..

[b0255] Ogawa S., Lee T.M., Kay A.R., Tank D.W. (1990). (1990) Brain magnetic resonance imaging with contrast dependent on blood oxygenation. PNAS.

[b0260] Ossenkoppele R., Schonhaut D.R., Schöll M., Lockhart S.N., Ayakta N., Baker S.L., O'Neil J.P., Janabi M., Lazaris A., Cantwell A., Vogel J., Santos M., Miller Z.A., Bettcher B.M., Vossel K.A., Kramer J.H., Gorno-Tempini M.L., Miller B.L., Jagust W.J., Rabinovici G.D. (2016). Tau PET patterns mirror clinical and neuroanatomical variability in Alzheimer's disease. Brain.

[b0265] Qiu T., Luo X., Shen Z., Huang P., Xu X., Zhou J., Zhang M. (2016). Alzheimer’s Disease Neuroimaging Initiative. Disrupted Brain Network in Progressive Mild Cognitive Impairment Measured by Eigenvector Centrality Mapping is Linked to Cognition and Cerebrospinal Fluid Biomarkers. J. Alzheimers Dis..

[b0270] Quevenco F.C., Preti M.G., van Bergen J.M., Hua J., Wyss M., Li X., Schreiner S.J., Steininger S.C., Meyer R., Meier I.B., Brickman A.M., Leh S.E., Gietl A.F., Buck A., Nitsch R.M., Pruessmann K.P., van Zijl P.C., Hock C., Van De Ville D., Unschuld P.G. (2017). Memory performance-related dynamic brain connectivity indicates pathological burden and genetic risk for Alzheimer's disease. Alzheimers Res. Ther..

[b0275] Quevenco F.C., Schreiner S.J., Preti M.G., van Bergen J.M.G., Kirchner T., Wyss M., Steininger S.C., Gietl A., Leh S.E., Buck A., Pruessmann K.P., Hock C., Nitsch R.M., Henning A., Van De Ville D., Unschuld P.G. (2019). GABA and glutamate moderate beta-amyloid related functional connectivity in cognitively unimpaired old-aged adults. Neuroimage Clin..

[b0280] Richiardi J., Gschwind M., Simioni S., Annoni J.M., Greco B., Hagmann P., Schluep M., Vuilleumier P., Van De Ville D. (2012). (2012) Classifying minimally disabled multiple sclerosis patients from resting state functional connectivity. Neuroimage.

[b0285] Risacher S.L., Kim S., Nho K., Foroud T., Shen L., Petersen R.C., Jack C.R., Beckett L.A., Aisen P.S., Koeppe R.A., Jagust W.J., Shaw L.M., Trojanowski J.Q., Weiner M.W., Saykin A.J. (2015). Alzheimer's Disease Neuroimaging Initiative (ADNI). APOE effect on Alzheimer's disease biomarkers in older adults with significant memory concern. Alzheimers Dement..

[b0290] Rombouts S.A.R.B., Damoiseaux J.S., Goekoop R., Barkhof F., Scheltens P., Smith S.M., Beckmann C.F. (2009). Model-free group analysis shows altered BOLD FMRI networks in dementia. Hum. Brain Mapp..

[b0295] Sanabria-Diaz G, Demonet JF, Rodriguez-Herreros B, Draganski B, Kherif F, Melie-Garcia L. Apolipoprotein E allele 4 effects on Single-Subject Gray Matter Networks in Mild Cognitive Impairment. Neuroimage Clin. 2021;32:102799. doi: 10.1016/j.nicl.2021.102799. Epub 2021 Aug 24. PMID: 34469849; PMCID: PMC8405842. 10.1016/j.nicl.2021.102799 PMC8405842 34469849

[b0300] Sanabria-Diaz G., Melie-Garcia L., Draganski B., Demonet J.F., Kherif F. (2021). Apolipoprotein E4 effects on topological brain network organization in mild cognitive impairment. Sci. Rep..

[b0305] Sanz-Arigita E.J., Schoonheim M.M., Damoiseaux J.S., Rombouts S.A., Maris E., Barkhof F., Scheltens P., Stam C.J. (2010). Loss of 'small-world' networks in Alzheimer's disease: graph analysis of FMRI resting-state functional connectivity. PLoS One.

[b0310] Scheltens P., De Strooper B., Kivipelto M., Holstege H., Chételat G., Teunissen C.E., Cummings J., van der Flier W.M. (2021). Alzheimer's disease. Lancet.

[b0315] Schreiner S.J., Van Bergen J.M.G., Gietl A.F., Buck A., Hock C., Pruessmann K.P., Henning A., Unschuld P.G. (2024). Gray matter gamma-hydroxy-butyric acid and glutamate reflect beta-amyloid burden at old age. Alzheimers Dement (Amst.)..

[b0320] Serrano-Pozo A., Das S., Hyman B.T. (2021). APOE and Alzheimer's disease: advances in genetics, pathophysiology, and therapeutic approaches. Lancet Neurol..

[b0325] Sheline Y.I., Morris J.C., Snyder A.Z., Price J.L., Yan Z., D'Angelo G., Liu C., Dixit S., Benzinger T., Fagan A., Goate A., Mintun M.A. (2010). APOE4 allele disrupts resting state fMRI connectivity in the absence of amyloid plaques or decreased CSF Aβ42. J. Neurosci..

[b0330] Sperling R.A., Dickerson B.C., Pihlajamaki M., Vannini P., LaViolette P.S., Vitolo O.V., Hedden T., Becker J.A., Rentz D.M., Selkoe D.J., Johnson K.A. (2010). (2010) Functional alterations in memory networks in early Alzheimer's disease. NeuroMol. Med..

[b0335] Su Y.Y., Zhang X.D., Schoepf U.J., Varga-Szemes A., Stubenrauch A., Liang X., Zheng L.J., Zheng G., Kong X., Xu Q., Wang S.J., Qi R.F., Lu G.M., Zhang L.J. (2017). Lower functional connectivity of default mode network in cognitively normal young adults with mutation of APP, presenilins and APOE ε4. Brain Imaging Behav..

[b0340] Tosto G, Monsell SE, Hawes SE, Mayeux R. Pattern of extrapyramidal signs in Alzheimer's disease. J Neurol. 2015 Nov;262(11):2548-56. doi: 10.1007/s00415-015-7886-1. Epub 2015 Sep 4. PMID: 26338814; PMCID: PMC4776751. 10.1007/s00415-015-7886-1 PMC4776751 26338814

[b0345] Verghese J, Wang C, Lipton RB, Holtzer R, Xue X. Quantitative gait dysfunction and risk of cognitive decline and dementia. J Neurol Neurosurg Psychiatry. 2007 Sep;78(9):929-35. doi: 10.1136/jnnp.2006.106914. Epub 2007 Jan 19. PMID: 17237140; PMCID: PMC1995159. 10.1136/jnnp.2006.106914 PMC1995159 17237140

[b0350] Vij S.G., Nomi J.S., Dajani D.R., Uddin L.Q. (2018). Evolution of spatial and temporal features of functional brain networks across the lifespan. Neuroimage.

[b0355] Wadhwani A.R., Affaneh A., Van Gulden S., Kessler J.A. (2019). Neuronal apolipoprotein E4 increases cell death and phosphorylated tau release in alzheimer disease. Ann. Neurol..

[b0360] Wang Z., Dai Z., Shu H., Liao X., Yue C., Liu D., Guo Q., He Y., Zhang Z. (2017). APOE genotype effects on intrinsic brain network connectivity in patients with amnestic mild cognitive impairment. Sci. Rep..

[b0365] Wang Y, Li Q, Yao L, He N, Tang Y, Chen L, Long F, Chen Y, Kemp GJ, Lui S, Li F. Shared and differing functional connectivity abnormalities of the default mode network in mild cognitive impairment and Alzheimer's disease. Cereb Cortex. 2024 Mar 1;34(3):bhae094. doi: 10.1093/cercor/bhae094. PMID: 38521993. 10.1093/cercor/bhae094 38521993

[b0370] Wang P., Zhou B., Yao H., Zhan Y., Zhang Z., Cui Y., Jiang T. (2015). Aberrant intra- and inter-network connectivity architectures in Alzheimer’s disease and mild cognitive impairment. Sci. Rep..

[b0375] Wen X, He H, Dong L, Chen J, Yang J, Guo H, Luo C, Yao D. Alterations of local functional connectivity in lifespan: A resting-state fMRI study. Brain Behav. 2020 Jul;10(7):e01652. doi: 10.1002/brb3.1652. Epub 2020 May 27. PMID: 32462815; PMCID: PMC7375100. 10.1002/brb3.1652 PMC7375100 32462815

[b0380] Wink AM, Tijms BM, Ten Kate M, Raspor E, de Munck JC, Altena E, Ecay-Torres M, Clerigue M, Estanga A, Garcia-Sebastian M, Izagirre A, Martinez-Lage Alvarez P, Villanua J, Barkhof F, Sanz-Arigita E. Functional brain network centrality is related to APOE genotype in cognitively normal elderly. Brain Behav. 2018 Sep;8(9):e01080. doi: 10.1002/brb3.1080. Epub 2018 Aug 22. PMID: 30136422; PMCID: PMC6160659. 10.1002/brb3.1080 PMC6160659 30136422

[b0385] Xu X., Chen P., Xiang Y., Xie Z., Yu Q., Zhou X., Wang P. (2022). Altered pattern analysis and identification of subjective cognitive decline based on morphological brain network. Front. Aging Neurosci..

[b0390] Yu M, Sporns O, Saykin AJ. (2021) The human connectome in Alzheimer disease - relationship to biomarkers and genetics. Nat Rev Neurol. 2021 Sep;17(9):545-563. doi: 10.1038/s41582-021-00529-1. Epub 2021 Jul 20. Erratum in: Nat Rev Neurol. 2021 Aug 11;: PMID: 34285392; PMCID: PMC8403643. 10.1038/s41582-021-00529-1 PMC8403643 34285392

[b0395] Yu J.-T., Tan L., Hardy J. (2014). Apolipoprotein E in Alzheimer’s dis- ease: An update. Annu. Rev. Neurosci..

[b0400] Zhang H., Jiang X., Ma L., Wei W., Li Z., Chang S., Wen J., Sun J., Li H. (2022). Role of Aβ in Alzheimer's-related synaptic dysfunction. Front. Cell Dev. Biol..

[b0405] Zheng L.J., Su Y.Y., Wang Y.F., Schoepf U.J., Varga-Szemes A., Pannell J., Liang X., Zheng G., Lu G.M., Yang G.F., Zhang L.J. (2018). Different hippocampus functional connectivity patterns in healthy young adults with mutations of APP/presenilin-1/2 and APOEε4. Mol. Neurobiol..

