# Supplementary material for: Functional network centrality indicates interactions between APOE4 and age across the clinical spectrum of AD
Source: Neuroimage Clin. 2024 Jun 24;43:103635. doi: 10.1016/j.nicl.2024.103635 (PMC11260379; doi:10.1016/j.nicl.2024.103635)
Supplement: Supplementary Data 1 [file mmc1.docx]

**Supplementary materials**


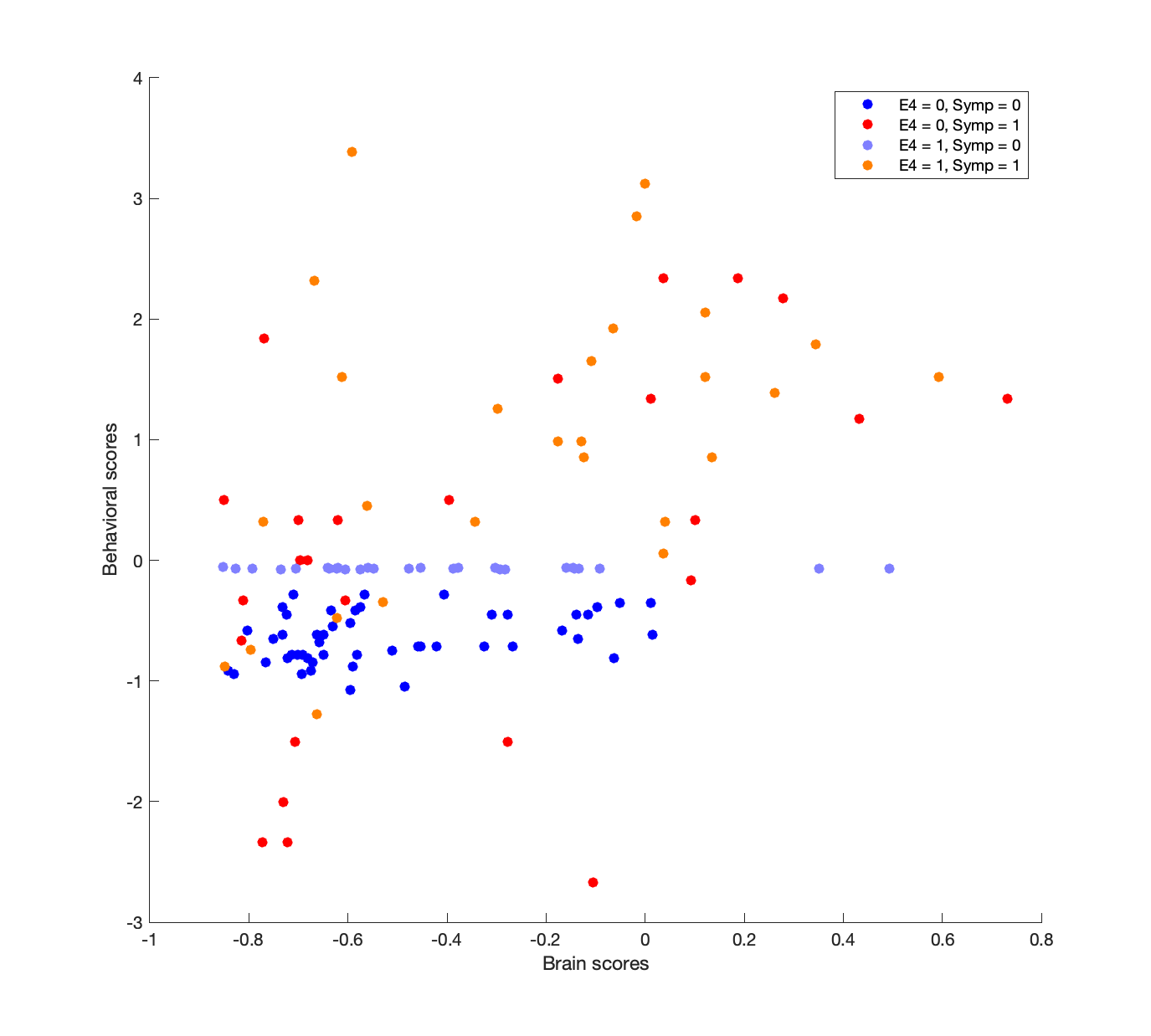


*Figure 7.* Behavioral scores for the first PLS component projected on brain scores and colored by APOE4 carrier status and by cognitive symptoms index. We observed that subjects with cognitive symptoms (red and purple) diverged from zero compared to other subjects (yellow and blue) and therefore are contributing the most to the brain pattern.

**
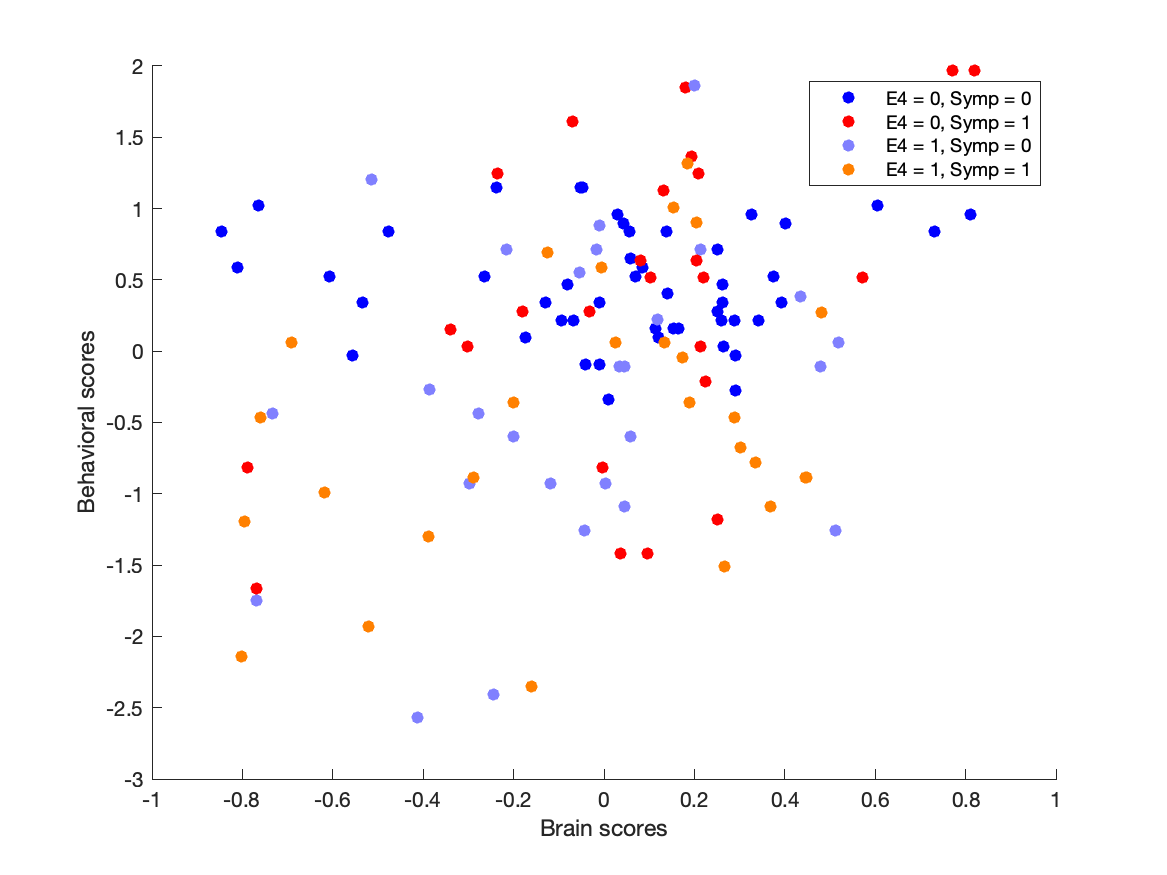
**

*Figure 8.* Behavioral scores for the second PLS component projected on brain scores and colored by APOE4 carrier status and by cognitive symptoms index. This graph shows the contribution of each subject in the brain pattern. We can see a small cluster of APOE4 carriers (purple and yellow dots in the bottom left) that seems to contribute more to the brain pattern than the other subjects (blue and red dots).
